# Letter from the Editor in Chief

**DOI:** 10.19102/icrm.2021.120106

**Published:** 2021-01-15

**Authors:** Moussa Mansour


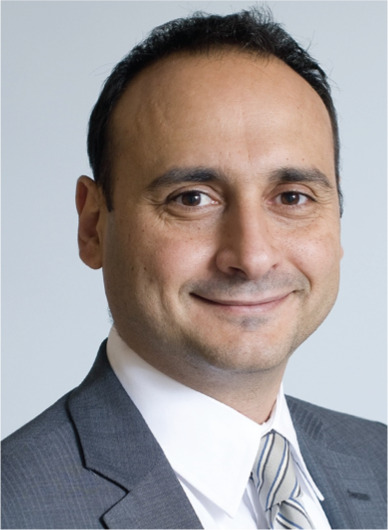


Dear Readers,

Despite the difficulties and challenges that occurred in medicine in 2020, cardiac electrophysiology continued to progress and many high-impact studies were published this past year. Below, I highlight a few that I believe will impact the field going forward.

The Prospective Randomized Comparison of Subcutaneous and Transvenous Implantable Cardioverter-defibrillator (ICD) Therapy (PRAETORIAN) trial was presented at the virtual HRS Science 2020 event and compared subcutaneous and transvenous ICDs in patients with indications for an ICD but not for pacing. The primary study endpoint was a composite of device-related complications and inappropriate shocks and 426 and 423 patients were randomized to the subcutaneous ICD and transvenous ICD groups, respectively. After a median follow-up period of 49 months, the primary endpoint was met and subcutaneous ICDs were found to be noninferior to transvenous ICDs in this investigation. This finding will likely result in an increased shift in clinical practice favoring subcutaneous ICDs for implantation.

Pulsed-field ablation has continued to make waves in 2020. At the Atrial Fibrillation (AF) Symposium in January 2020, Reddy al. presented data from a first-in-human trial of a lattice-tip, temperature-controlled radiofrequency ablation catheter, focusing on pulmonary vein isolation (PVI) outcomes and linear lesion durability. A total of 60 patients were treated at three European centers using this novel technology; some underwent tricuspid and mitral isthmus ablation in addition to PVI. Two important findings arose from this investigation: first, the rate of durable PVI at remapping study was 96.3%, while, at 291 ± 106 days of follow-up, the 12-month Kaplan–Meier estimate for freedom from all atrial arrhythmias was 94.4% ± 3.2%. The same group reported the results of the PersAFOne study as well, which examined the use of pulsed-field ablation to treat persistent AF with PVI plus posterior wall ablation. Here, 15 patients with persistent AF were enrolled in two European centers and underwent PVI and posterior left atrial wall ablation. This pilot study supported the view that pulsed-field ablation is a promising modality and facilitates ultrafast ablation of AF with no major complications.

Also during HRS Science 2020, the acute results of the PULSED AF study, a nonrandomized, prospective, multicenter, global premarket clinical study performed in Australia, Canada, the United States, and Europe, were presented. This trial evaluated the PulseSelect system (Medtronic, Minneapolis, MN, USA), which delivers pulsed electric fields through a circular multielectrode array catheter for PVI. Ablation in patients with either paroxysmal or persistent AF led to acute electrical isolation in 100% of this population without tamponades, strokes, or phrenic nerve injuries. Upon conclusion, data on the rate of arrhythmia-free survival at 12 months along with prespecified secondary and ancillary endpoints such as procedural outcomes, quality of life, and arrhythmic symptoms are anticipated.

Two large multicenter studies on the ablation of persistent AF were presented in 2020. The STOP Persistent AF global prospective multicenter study was presented at the 2020 AF Symposium, with Calkins et al. offering 12-month efficacy and safety results. This study found that cryoballoon ablation using the Arctic Front Advance™ system (Medtronic) is safe and effective in the ablation of persistent AF. The 12-month efficacy rate of ablation using this technology was 55%. The study confirmed the hypothesis that, to achieve higher success rates when treating persistent AF, adjunctive ablation in addition to PVI will likely be required.

Separately, the PRECEPT study, the first prospective, multicenter investigational device exemption study designed to evaluate the safety and effectiveness of radiofrequency catheter ablation in patients with persistent AF, was presented at 2020 HRS Science. The primary effectiveness endpoint was freedom from documented recurrence of atrial flutter/atrial tachycardia episodes of 30 seconds or longer and from the following additional five failure modes: acute procedural failure, use of a nonstudy catheter, repeat procedures, use of new/higher-dose antiarrhythmic drugs, and surgical AF ablation. At 15 months postprocedure, the primary effectiveness endpoint was achieved in 62% of participants. Moreover, 86.1% of patients had freedom from the need for repeat ablation, while 80.4% remained free from any documented, symptomatic atrial arrhythmias. Based on the results of these two studies, the Arctic Front Advance cryoablation system and the ThermoCool SmartTouch SF ablation catheter (Biosense Webster, Diamond Bar, CA, USA), respectively, were granted approval by the United States Food and Drug Administration (FDA) for the treatment of persistent AF.

A few studies also demonstrated the benefit of early intervention in AF. At the European Society for Cardiology 2020 virtual meeting, the EAST-AFNET 4 study was presented as a late-breaking clinical trial with simultaneous publication in *The New England Journal of Medicine*,^1^ where a total of 2,789 patients with early AF and other cardiovascular conditions were randomized to either early rhythm control or rate control. Two primary endpoints were considered: (1) a composite of death from cardiovascular causes, stroke, or hospitalization with worsening of heart failure or acute coronary syndrome and (2) the number of nights spent in the hospital. The primary safety outcome was a composite of death, stroke, or other treatment-related serious adverse events. During follow-up, the early rhythm-control strategy proved superior regarding both primary points. Interestingly, during the first two years of follow-up, only 19% of patients in the rhythm-control group underwent ablation, while antiarrhythmic medications were given to the remaining majority. There was no significant difference in the rate of adverse events between the two groups.

Also presented at the same meeting, STOP AF First compared cryoballoon ablation and antiarrhythmic drug therapy as first-line treatments in patients with paroxysmal AF. A total of 225 patients were enrolled and followed for two years; freedom from AF was ultimately achieved in 75% undergoing ablation as compared with in 45% in the medication group. Most importantly, ablation was associated with a very low rate of complications (1.9%).

Finally, results of the prospective nonrandomized PINNACLE FLX trial, which enrolled 400 patients, were presented by Dr. Doshi at 2020 HRS Science. Notably, the study procedure was associated with a very low adverse event rate of 0.5% and a 100% rate of effective left atrial appendage closure at 12 months postprocedure. This is an important study demonstrating the safety and efficacy of the new-generation WATCHMAN FLX left atrial appendage closure device (Boston Scientific, Natick, MA, USA) and, based on its findings, the device was approved by the FDA.

Best wishes for a happy and healthy new year. I hope that you enjoy this issue of *The Journal of Innovations in Cardiac Rhythm Management.*

Sincerely,


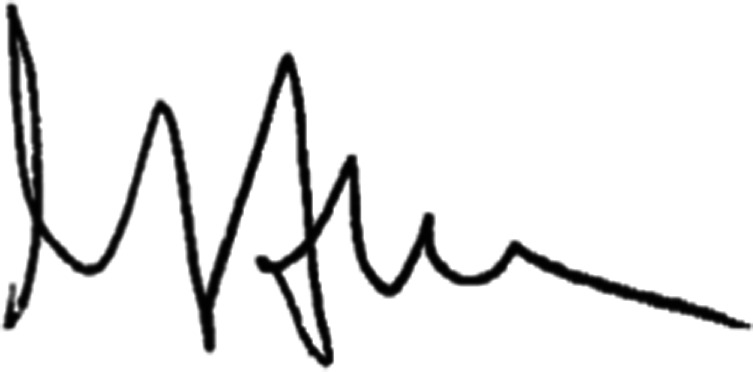


Moussa Mansour, MD, FHRS, FACC

Editor in Chief

The Journal of Innovations in Cardiac Rhythm Management

MMansour@InnovationsInCRM.com

Director, Atrial Fibrillation Program

Jeremy Ruskin and Dan Starks Endowed Chair in Cardiology

Massachusetts General Hospital

Boston, MA 02114
